# Comparative Analysis of Pressure Drop (Δp): Hemodynamic Performance of Three Commercial Oxygenators During Cardiopulmonary Bypass

**DOI:** 10.1111/aor.70088

**Published:** 2026-01-19

**Authors:** Jan Turra, Martina Habermehl, Thomas Schiepp, Peter Rose, Berthold Klein, Benjamin Scheerer, Matthias Karck, Christoph Lichtenstern, Riesterer David Max, Dania Fischer

**Affiliations:** ^1^ Department of Perfusiologie, Cardiac Surgery University Hospital Heidelberg Heidelberg Germany; ^2^ Department of Perfusiologie, Cardia Surgery University Hospital German Heart Centre Munich Munich Germany; ^3^ Hochschule Furtwangen Villingen‐Schwenningen Germany; ^4^ Department of Cardiac Surgery University Hospital Heidelberg Heidelberg Germany; ^5^ Department of Anesthesiology University Hospital Heidelberg Heidelberg Germany

**Keywords:** cardiopulmonary bypass, heart‐lung‐machine, hemolysis, influencing factors, oxygenator pressure, Δp

## Abstract

**Backround:**

The membrane oxygenator is a critical component of extracorporeal circulation (ECC), whose performance directly depends on its structural architecture. Differences in fiber geometry and winding patterns lead to variable hemodynamic pressure conditions. The resulting pressure drop Delta p (Δp) across the oxygenator module is a decisive indicator for system load and the risk of blood trauma. The aim of this study is the comparative analysis of the Δp of three commercial oxygenators: Medtronic Affinity Fusion, Eurosets A.L. ONE Plus, and LivaNova Inspire 8F, calculated from the difference between inlet and outlet pressure.

**Methods:**

This prospective observational study included 60 patients divided into three groups of 20 patients each, based on the type of oxygenator used in their perfusion circuit.

**Results:**

The results of the study show that the Δp value was significantly higher for the Inspire 8F compared to the Affinity Fusion and A.L. ONE Plus oxygenators. Between measurement points T2 and T11, the highest Δp was 116 ± 56 mmHg for the Medtronic group, 123 ± 26 mmHg for the Eurosets group, and 168 ± 33 mmHg for the Inspire 8F group. No significant differences in Δp were observed between the Affinity Fusion and the A.L. ONE Plus at any measurement point.

**Conclusion:**

The results of the study show that the Inspire has the highest Δp of all the oxygenators examined in this study. In addition to potential influencing factors, the value is also dependent on the internal structure and the surface properties of the oxygenators. However, the present study does not help to clarify the effects of the pressure before and after the oxygenator or the Δp itself on hemolysis and subsequent clinical parameters associated with the use of different commercially available oxygenators.

## Graphical abstract

In this prospective study, a total of 60 patients were divided into three groups of 20 to compare the pressure drop (Δp) across three different oxygenators during cardiopulmonary bypass. The aim of this comparison was to enable evidence‐based optimization of the heart‐lung machine setup in order to minimize potential influencing factors and thereby improve patient outcomes in cardiac surgery.

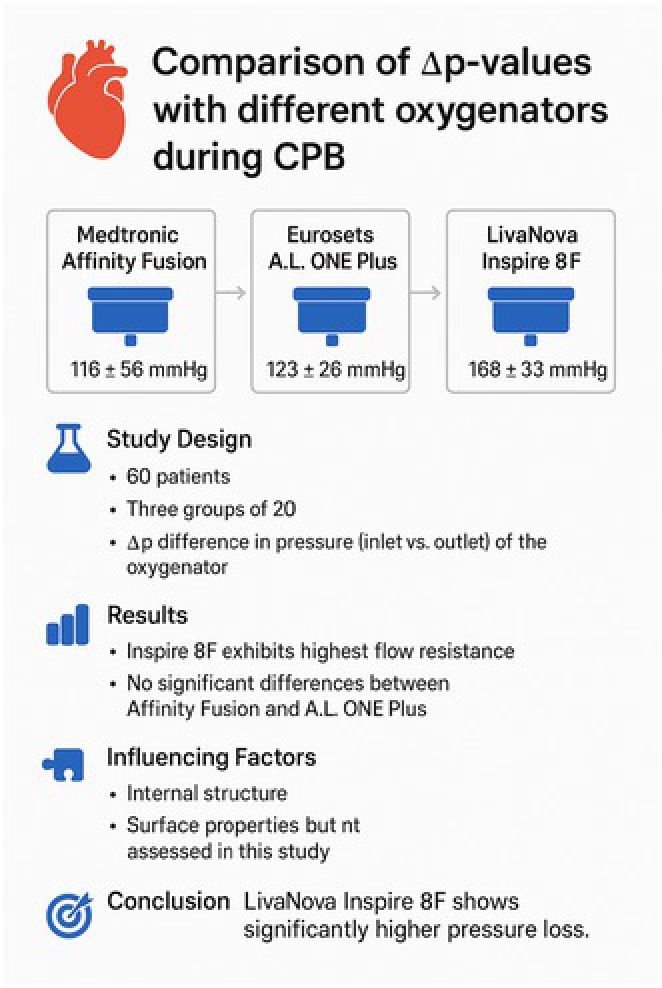

## 1 Introduction

Oxygenators are critical components of Cardiopulmonary Bypass (CPB) circuits, as their structural architecture directly influences the mechanical forces exerted on the blood, playing a significant role in hemolysis [1, 2, 3]. Shear stress is generated as blood is pumped through the fine fibers and narrow pathways within the oxygenator [4]. A high‐pressure difference, or Δp, across the oxygenator usually indicates greater resistance to blood flow, which can force cells through smaller spaces and increase mechanical trauma [5, 6]. Furthermore, operating oxygenators outside their optimal flow range (e.g., too high) can create turbulence and microbubbles, leading to Red Blood Cell (RBC) damage [7].

The membrane oxygenator must achieve comparable gas exchange performance within its small 1.3–2.5 m^2^ surface area during the ECC compared to the approximately 100 m^2^ surface area of the pulmonary alveoli [8, 9]. This efficiency is partly achieved through higher pressure ratios between the venous side and the gas side [8, 9]. However, this leads to high pressures, up to 550 mmHg, occurring at the oxygenator inlet [7, 10, 11]. When these transoxygenator pressures (or Δp) become too high, hemolysis can result [7, 10, 11]. While De Somer's 2013 study could not establish a linear relationship between the pressure drop and hemolysis, it reported significant damage to platelets and erythrocytes, followed by subsequent activation of coagulation cascades [10]. Another study by Fisher et al. found an abnormal pressure gradient in one of 230 cases and showed a clear difference in Δp between the investigated oxygenators [12]. Critically, the relationships between oxygenator design, hemolysis, and activation of the coagulation cascade remain not finally clarified [10].

In this prospective, observational study, we aim to compare three commercially available oxygenators in terms of their pressure behavior and the respective influencing parameters. By precisely analyzing the pressure profiles Δp, we seek to identify pressure fluctuations and their potential correlations with routinely monitored ECC parameters, thereby providing crucial hemodynamic baseline data.

## 2 Methods

### 2.1 Study Design and Settings

This prospective observational study was planned and performed at the Heidelberg University Hospital, Germany. Patients were recruited from September to December 2024. The study was performed in conformance with the ethical standards of the Declaration of Helsinki and its later amendments.

### 2.2 Ethics

The study protocol was approved by the Institutional Ethical Committee of the Medical Faculty of Heidelberg University (reference number: S‐481/2024).

### 2.3 Participants

This prospective observational study included 60 patients, divided into three groups of 20 each, based on the type of oxygenator used in their perfusion circuit. The decision for the specific oxygenator to be used for CPB was made independently of this study. Inclusion criteria were that all patients in this study underwent elective isolated CABG procedures under standardized extracorporeal circulation conditions, central aorto‐venous single cannulation, and age ≥ 18 years. Exclusion criteria were emergency interventions and interventions involving re‐operation (e.g., due to post‐operative bleeding) within the first 24 h post‐op.

### 2.4 Study Protocol Anesthesia and Surgery

All procedures were performed in general anesthesia. The treating anesthesiologist determined ventilation and fluid management before ECC. An inhalational agent (sevoflurane) combined with sufentanil was used for anesthesia maintenance. All patients received a midline sternotomy and a central cannulation in preparation for CPB. The return cannula was connected to the arterial line (3/8 × 3/32‐in. tubing) and the drainage cannula was connected to the venous line (1/2 × 3/32‐in. tubing) of the HLM. All patients received Bretschneider cardioplegia (Custodiol) as the myocardial protection strategy. The volume administered ranged between 1 and 2 L, depending on body weight and surgical duration.

### 2.5 Study Protocol Heart‐Lung‐Machine

**FIGURE 1:**
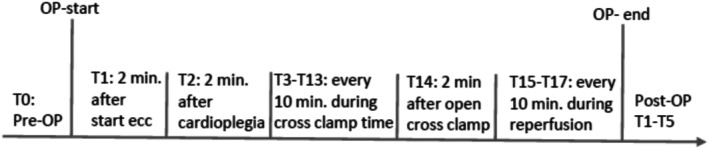
Timeline for the different measurement times.

All ECC procedures were run according to standard perfusion regime. The three oxygenators examined were: Medtronic Affinity Fusion (rated for a blood flow of up to 7 L/min, maximum arterial pressure 750 mmHg, membrane surface 2.5m^2^), Eurosets A.L. ONE Plus (rated for a blood flow of up to 7 L/min, maximum arterial pressure 750 mmHg, membrane surface 1.65 m^2^) and LivaNova Inspire 8F (rated for a blood flow of up to 8 L/min, maximum arterial pressure 750 mmHg, membrane surface 1.65). The extracorporeal circulation was performed using a Stöckert S5 heart‐lung machine (LivaNova, formerly Sorin Group) equipped with a roller pump system. The system was primed with 1000 mL of balanced electrolyte solution and 10 000 IU of unfractionated heparin. The pre‐bypass filter was briefly disconnected in order to be able to record the initial pressure values on the oxygenator at the patient‐specific cardiac output (CO) (measurement time T0). After all the grafts required for the operation were prepared, the anesthetist administered heparin to the patient at a concentration of 400 i.U. per kg body weight with the aim to reach an activated clotting time (ACT) of at least 450 s. After connecting the HLM tubes to the cannulas, the ECC was started. Over a period of about 2 min, the flow of the arterial pump was slowly increased and at the same time the venous throttle was progessively opened. Two minutes after the start of the ECC, the first data recording (T1) and the first arterial blood gas analysis was taken. The data collected included arterial blood gas analysis, pre‐ and post‐oxygenator pressure, Δp, CO and arterial temperature. The second data recording (T2) was taken 2 min after the application of cardioplegia. During the subsequent aortic clamping time, data was documented every 10 min (T3–T13). If the use of a hemofilter was necessary during the operation, it was briefly disconnected for data recording. As soon as the aorta was unclamped and the reperfusion phase began, data was recorded again after 2 min (T14) and then continued at 10 min's intervals until the ECC was terminated (T15–T17) as the timeline shows in Figure 1. If the patient became unstable and ECC had to be restarted after weaning, data recording was discontinued at that point. After completion of surgery and transfer of the patient to the intensive care unit, the first laboratory was taken immediately.

All procedures were performed without the use of vacuum‐assisted venous drainage (VAVD). Roller pump occlusion was carefully checked and standardized prior to each procedure. Suction systems and circuit components remained consistent throughout all cases to minimize variability in hemolysis risk.

### 2.6 Measurements

RAPIDPoint 500 System from Siemens Healthineers (Munich/Germany) was used for routine blood gas analysis during ECC.

### 2.7 Primary Outcome

Pressure at the inlet and outlet of the oxygenator, and the Δp across the different oxygenators during HLM use in ECC.

### 2.8 Secondary Outcomes

Blood temperature, blood pressure, CO, and hemoglobin were also monitored.

### 2.9 Statistical Methods

Statistical analysis was performed using ANOVA (F‐test) to compare three independent groups. The significance level was set at 5% (*α* = 0.05). The null hypothesis was rejected if the test statistic F exceeded the critical value Fk, corresponding to the (1–*α*) quantile of the F‐distribution. In addition, *p*‐values were calculated and compared to the significance level. If the ANOVA detected a significant difference among the three groups, pairwise comparisons between groups were performed using two‐sample t‐tests at the respective measurement times. To account for multiple testing and to control the family‐wise error rate, the significance level was adjusted using the Bonferroni correction. Accordingly, the significance level for each individual *t*‐test was set to 1.667% (*α* = 0.01667), ensuring that the overall significance level across the three comparisons remained at 5%. The data was analyzed using quantile‐quantile plotting (Q‐Q plotting).

## 3 Results

Table 1 shows the demographic data of the three groups. There is a significant difference in body weight and CO. All other demographic data are not significant.

**TABLE 1:** Demographic patient data of the three comparison groups.

		Medtronic	Euroset	Inspire	
Number	(*n*)	20	20	20	
Gender	Female	2 (10%)	6 (30%)	0 (0%)	
	Male	18 (90%)	14 (70%)	20 (100%)	
Age	[Years]	64 ± 11	67 ± 8	70 ± 9	ns
Body size	[cm]	175 ± 7	172 ± 8	176 ± 9	ns
Body weight	[kg]	91 ± 18	79 ± 13	81 ± 12	*p* < 0.05 (*p* = 0.02)
BSA	[m^2^]	2.05 ± 0.20	1.92 ± 0.18	1.98 ± 0.19	ns
CO	[l/min]	5.13 ± 0.49	4.75 ± 0.44	4.95 ± 0.47	*p* < 0.05 (*p* = 0.045)
Bypasstime	[Minutes]	103 ± 25	101 ± 28	109 ± 28	ns
Cross clamp time	[Minutes]	68 ± 25	66 ± 20	72 ± 20	ns
Reperfusion	[Minutes]	24 ± 6	25 ± 10	29 ± 13	ns

*Note:* Mean values ± standard error. *p* < 0.05, significant.

Abbreviations: ns, not significant.

### 3.1 Pressure Gradient Across the Oxygenator

**FIGURE 2:**
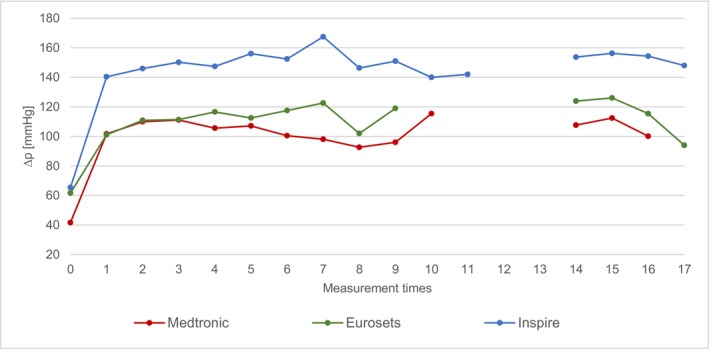
Comparison of mean values Δp. [Color figure can be viewed at wileyonlinelibrary.com]

The mean values of all intraoperative measurements (T0–T17) are shown graphically in Figure 2.

At time T0, the lowest ∆p is measured in the Medtronic group (mean value 42 ± 17 mmHg), while the Eurosets group is at 62 ± 22 mmHg and Inspire at 65 ± 10 mmHg. T1–T10 represent the values during ECC start and aortic clamping time. No data are available for the Eurosets group for the time points T10 and T11, and no data are available for Medtronic for T11 and T17. At measurement T1, the Δp for the Medtronic group shows a mean value of 102 ± 25 mmHg, for Eurosets 101 ± 19 mmHg and for the Inspire group 140 ± 30 mmHg. During the time points T2–T11, the highest Δp for the Medtronic group is 116 ± 56 mmHg and the lowest is 93 ± 20 mmHg. Eurosets has the highest Δp at 123 ± 26 mmHg and the lowest at 102 ± 24 mmHg. For the Inspire, the highest Δp is 168 ± 33 mmHg and the lowest 140 mmHg (standard deviation cannot be specified as only one measured value is available for this time point). At time T14, the Medtronic has a mean value of 108 ± 26 mmHg, the Eurosets of 124 ± 33 mmHg and the Inspire of 154 ± 38 mmHg. At the measurement times T1–T8 and T14–T16, there are significant differences in the Δp between Medtronic—Inspire and Eurosets—Inspire. With the exception of the measurement times T8, T15 and T16, there are only significant differences in the Δp between Medtronic—Inspire.

**FIGURE 3:**
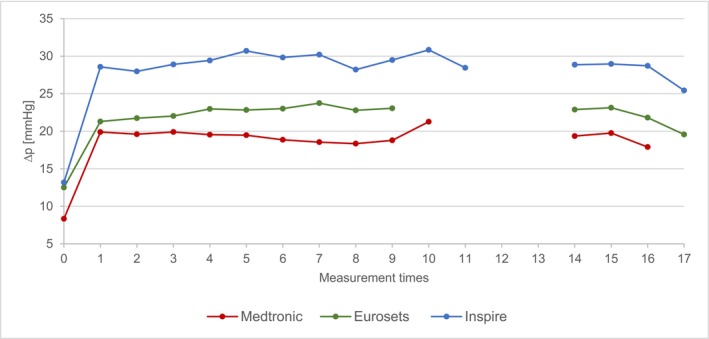
Mean value comparison Δp normalized. [Color figure can be viewed at wileyonlinelibrary.com]

Figure 3 shows the Δp of the three oxygenators normalized to a flow of 1 L/min: Standardized to a flow rate of 1 L/min, the pressure differences of the Medtronic range from 21 ± 5 mmHg to 18 ± 2 mmHg over the entire recording period. With the Eurosets, the highest value is 24 ± 4 mmHg and the lowest 20 ± 2 mmHg. The Inspire has a maximum value of 30 ± 2 mmHg and a minimum of 28 ± 3 mmHg.

Using the ANOVA test, there is a significant difference in the Δp between all three comparison groups for the measurement times T1–T16.

### 3.2 Cardiac Output

**FIGURE 4:**
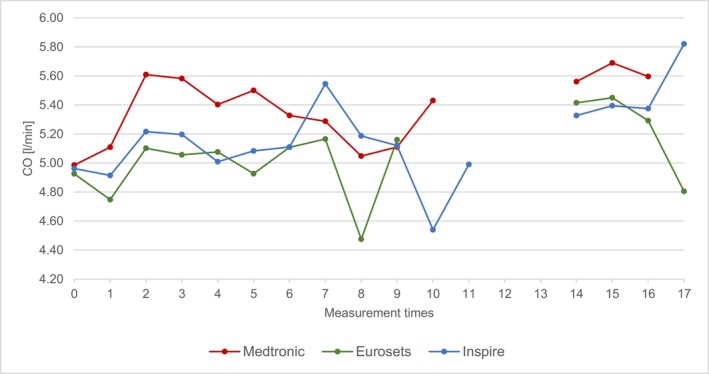
Mean value comparison CO. [Color figure can be viewed at wileyonlinelibrary.com]

The mean values of the CO of the three groups are shown in Figure 4 as follows:

Across all three groups, the mean value during the entire measurement period was between 4.48–5.82 L/min. The CO at time T1 is between 4.75 ± 0.45 L/min for Eurosets and 5.11 ± 0.68 L/min for Medtronic and 4.91 ± 0.73 L/min for Inspire. T16 shows 5.29 ± 0.80 L/min for Eurosets, 5.37 ± 1.07 L/min for Inspire and 5.60 ± 0.64 L/min for Medtronic. The CO showed a significant difference at time T3 between Medtronic‐Euroset *p*‐value = 0.00775.

### 3.3 Hemoglobin

**FIGURE 5:**
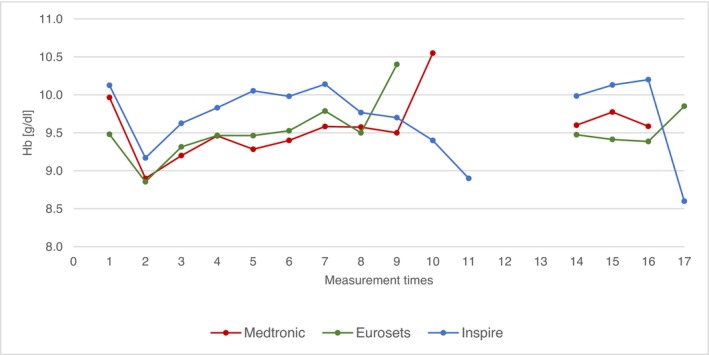
Mean value comparison Hb. [Color figure can be viewed at wileyonlinelibrary.com]

Figure 5 shows the mean values of hemoglonim (Hb). The mean Hb values were remained comparable across the three groups during the entire measurement period.

Blood transfusion was transfused in all three groups. The Medtronic group recorded an average of 0.9 blood transfusions per patient, the Euroset group 1 blood transfusion per patient and the Inspire group 0.9 EC blood transfusion. No significant difference was found between the groups with regard to blood transfusion administration.

### 3.4 Mean Arterial Blood Pressure

**FIGURE 6:**
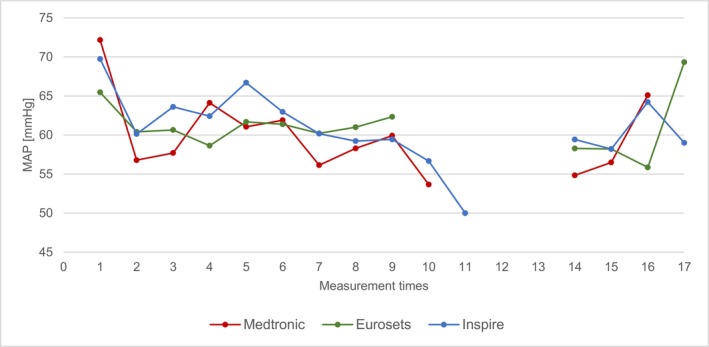
Mean value comparison MAP. [Color figure can be viewed at wileyonlinelibrary.com]

Figure 6 shows the mean values of the mean arterial blood pressure (MAP) over the measurement times. These did not differ between the three groups over the entire measurement period.

### 3.5 Arterial Temperature

**FIGURE 7:**
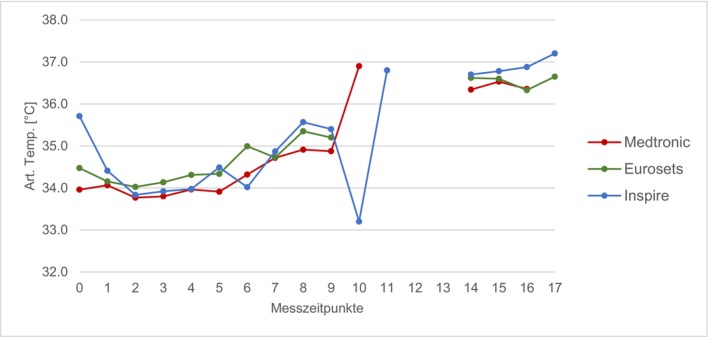
Comparison of mean arterial temperature. [Color figure can be viewed at wileyonlinelibrary.com]

Figure 7 shows the mean values of the arterial temperature over the measurement period: The course of the arterial temperature shows the patient's cooling phase immediately after the start of the ECC (T1 and T2) and the rise in temperature during the rewarming phase. With the exception of time T10, the mean values of the three comparison groups were not more than 1°C apart at any time during the ECC. The mean value of the arterial temperature of the Inspire droped to 33.2°C at T10 (from 35.4°C at T9) and rose again to 36.8°C at T11 (standard deviation cannot be specified as only one measured value is available in each case). The arterial temperature between T14 and T18 was equal.

## 4 Discussion

The primary objective of this study was the comparative analysis of the hemodynamic performance of three distinct commercial oxygenators by quantifying the Δp under clinical Cardiopulmonary Bypass (CPB) conditions.

### 4.1 Hemodynamic Performance and Blood Trauma Potential

The data reveal a statistically significant difference in Δp across the devices, with the LivaNova Inspire 8F exhibiting a consistently and significantly higher Δp than the Medtronic Affinity Fusion and Eurosets A.L. ONE Plus. Specifically, the Δp of the Inspire was, on average, 32% higher than the Eurosets and 46% higher than the Medtronic across the evaluated time periods (e.g., 140 ± 30 mmHg to 168 ± 33 mmHg for Inspire versus 93 ± 20 mmHg to 112 ± 29 mmHg for Medtronic). This hemodynamic variation is strongly corroborated by previous literature, which also identified the Inspire family as producing higher pressure gradients compared to other market competitors [5, 13].

The observation of a higher Δp directly correlates with a greater mechanical load imposed on the blood components. This increased resistance necessitates a higher driving pressure, which in turn leads to elevated shear stress within the device's complex fiber matrix. Mechanically, increased shear stress—a consequence of forcing flow through narrow or convoluted pathways—is the primary mechanism linking high Δp to the pathophysiology of hemolysis and platelet activation [14]. Damage to RBCs results in hemoglobin release, while excessive shear can also activate platelets and trigger the coagulation cascade [14]. Therefore, the significant Δp variation identified in the Inspire oxygenator provides a crucial mechanical baseline, strongly suggesting that this device may induce a greater burden of sub‐lethal and lethal trauma on blood components during routine CPB.

These pressure differences are primarily attributed to the inherent material properties, fiber density, and flow characteristics dictated by the oxygenator's structural design, a conclusion supported by normalizing the Δp to a standard flow rate of 1 L/min, which maintained the highly significant distinctions between the devices.

### 4.2 Patient Comparability and Systemic Influences

A crucial aspect of this comparison is the rigorous demonstration of comparability across the three observational groups. The analysis confirmed that no significant differences were found between the groups regarding core procedural factors (bypass time, aortic clamping time, reperfusion time) or key systemic influences.

Analysis of routine ECC parameters—including CO, Hb, MAP, and arterial temperature—revealed that these factors did not exert a direct, confounding influence on the measured Δp differences. For instance, the Δp remained constant despite simultaneous, expected fluctuations in CO and Hb during events like cardioplegia administration. This stability suggests that the differences in Δp are primarily device‐dependent (i.e., structural architecture) and not driven by transient patient or perfusion conditions, thereby affirming the robustness of the device comparison [5, 6, 13].

### 4.3 Catastrophic Oxygenator Failure and Δp

While modern oxygenators are highly reliable, the potential for acute, catastrophic failure due to hemodynamic compromise remains a critical safety concern during CPB [15]. An excessively or rapidly increasing Δp serves as the primary early indicator of this risk [16]. A steep rise in Δp often signals progressive fiber bundle obstruction, typically due to thrombosis or the accumulation of microaggregates, which significantly increases resistance within the module. This obstruction necessitates critically high inlet pressures to maintain the prescribed blood flow, raising the risk of severe mechanical damage, potential membrane rupture, or complete flow stagnation within the device [17]. Such an acute, uncontrolled rise in system load constitutes catastrophic failure, demanding immediate, unscheduled replacement of the oxygenator unit during the ongoing CPB run. This emergency procedure introduces significant complexity, operational delay, and severe added risk to the patient, underscoring the necessity of selecting devices with the most favorable baseline Δp profile.

## 5 Conclusion and Clinical Relevance

The findings confirm the null hypothesis and demonstrate that the LivaNova Inspire 8F exhibits a significantly higher Δp than its comparators under routine CPB conditions. While this elevated Δp is strongly linked to a theoretical increase in mechanical stress and potential for blood trauma, the present study does not include direct measurements of hemolysis or platelet function. Therefore, the clinical significance of the observed elevated Δp remains to be fully elucidated and necessitates further investigation, particularly through powered, prospective studies designed to correlate these hemodynamic markers with direct hemolytic effects and subsequent clinical outcomes.

## 6 Limitation

The study was designed as an exploratory, prospective observational trial and was not powered to detect statistically significant differences between subgroups based on demographic or clinical baseline parameters. A formal sample size calculation was not performed, as the primary aim was to assess pressure gradients across different oxygenators under routine clinical conditions, rather than to establish definitive comparative efficacy. The findings should therefore be interpreted with caution, and future studies with larger sample sizes and matched cohorts are warranted to confirm these results and minimize potential selection bias.

## Author Contributions

J.T.: Concept/design, preparation of the ethics application, data analysis/interpretation, writing drafting article, critical revision of article, approval of article, statistics, funding secured by, data collection, review and editing, supervision; M.H.: data analysis/interpretation, writing drafting article, critical revision of article, approval of article, statistics, funding secured by, data collection; T.S.: critical revision of article; P.R.: preparation of the ethics application, critical revision of article; B.K.: critical revision of article; M.K.: critical revision of article; C.L.: critical revision of article; R.D.M.: critical revision of article, data analysis/interpretation; D.F.: data analysis/interpretation, writing drafting article, critical revision of article, approval of article, funding secured by, supervision.

## Conflicts of Interest

The authors declare no conflicts of interest.

## Data Availability

The data that support the findings of this study are available from the corresponding author upon reasonable request.
